# Open total dislocation of ankle joint without fractures: A case report

**DOI:** 10.1097/MD.0000000000026247

**Published:** 2021-06-04

**Authors:** Yu-lei Chi, Xu Gao, Ying-jie Xu, Xian-min Bu, Liang Han, Xu Zhang, Long-fei Gao, Rong-hua Tian, Hai-bin Wang, Bin Wu

**Affiliations:** a Department of Clinical Medicine, Jining Medical University, Jining City; b Department of Qingdao Medical College, Qingdao University, Qingdao City; c Department of Pathology, Jining No. 1 People's Hospital; d Department of Orthopedics, Affiliated Hospital of Jining Medical University, Jining City, Shandong Province, China.

**Keywords:** ankle joint, fractures, ligaments, total dislocation

## Abstract

**Rationale:**

Open total dislocation of ankle joint is rare and often caused by high-energy injury. The present study describes a patient with open total lateral dislocation of ankle joint without fractures and obtained a satisfactory clinical result following early debridement and irrigation, one-stage repairment of ligaments, and plaster external fixation.

**Patient concerns:**

The patient, a 45-year-old male, complained of right foot pain with bleeding and limited motion. Physical examination showed a 15-cm open wound at the medial ankle region, with soft tissues impaired and ankle bones exposed. The 3 dimensional reconstruction computed tomography (CT) examination showed an open total dislocation of ankle joint without concomitant fractures.

**Diagnoses:**

open total lateral dislocation of ankle joint without fractures

**Interventions:**

Early modern wound care including thorough debridement and irrigation on the wound was performed to remove contaminated soft tissues. Subsequently, the dislocated ankle joint was reduced by hand and the medial and lateral collateral ligaments were repaired using wire anchors.

**Outcomes:**

The medial wound healed at 2 weeks after surgery, and several common complications such as infection and skin necrosis did not occur. The last follow-up showed a good range of metatarsal flexion and extension of the injured foot, and obvious signs of traumatic arthritis were not observed. According to Kaikkonen ankle function score, the patient was assessed with 90 points.

**Lessons:**

For open total dislocation of ankle joint, early treatment should focus on debridement and irrigation, reduction and fixation of the dislocated ankle, protection of the weak soft tissues, and stable external fixation to promote wound healing and reduce the incidence of related complications.

## 1 Introduction

Ankle joint, a load-bearing joint, is a complex hinge system which is essential for human movement. Total dislocation of ankle joint is first reported by Peraire in 1913,^[1]^ and is usually attributed to a high-energy injury. Comparatively, isolated ankle joint dislocation without concomitant fractures of medial or lateral malleolus is uncommon in the clinical practice.^[2]^ In the present study, we report a case of open total dislocation of ankle joint without fractures in a patient who obtained a satisfactory clinical result following early debridement and irrigation, one-stage repairment of ligaments, and plaster external fixation.

## 2 Case report

**Figure 1:**
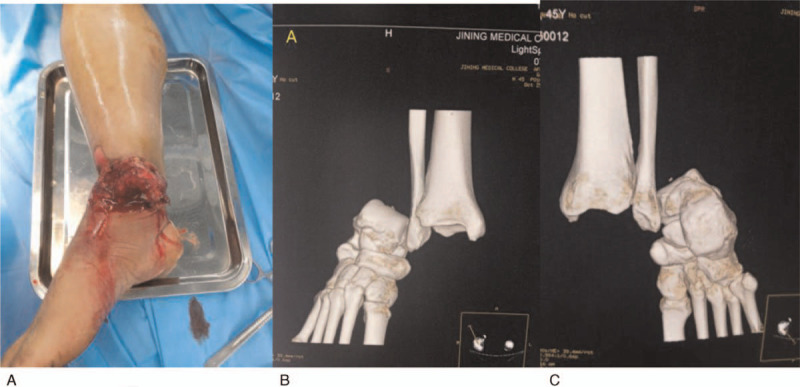
(A) The patient, a 45-year-old male, had an open wound with ankle bone and articular cartilage exposed; (B, C) Emergency three-dimensional CT examination of the ankle joint showed total ankle joint dislocation without fractures.

**Figure 2:**
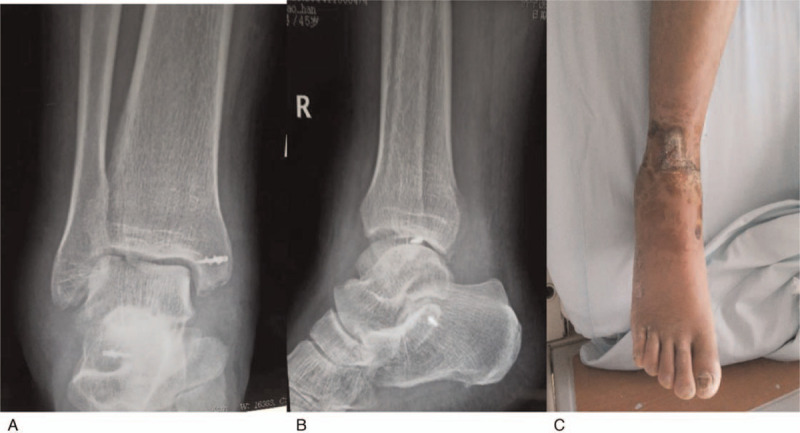
(A, B) Fluoroscopy showed an normal anatomy of ankle joint; (C) The wound healed 2 weeks after operation, and there were no complications such as infection and skin necrosis.

A 45-year-old male, a coal miner, suffered an impact injury and then was admitted emergently to our institution with right foot pain, bleeding, limited motion and deformity. Physical examination found a 15-cm transverse lacerated wound at the medial ankle region with ankle bone and articular cartilage exposed and surrounding soft tissues injured seriously (Fig. 1a). Palpation of distal dorsalis pedis artery pulse was weak, with plantar foot sensation reduced. The three-dimensional reconstruction computed tomography (CT) examination revealed total dislocation of ankle joint without concomitant fractures (Fig. 1b,c). Immediately, a manipulative reduction was attempted but failed. Subsequently, the patient was taken into emergency operating room and subject to emergent surgery under general anesthesia. Thorough debridement and irrigation on the medial wounds were performed first to remove contaminated and inactivated soft tissues, and we found the lateral and medial collateral ligaments were torn completely. After wound debridement, the dislocated ankle joint was reduced by squeezing the talus inward. Clinical examination further found the restored ankle joint was unstable, and then the medial and lateral ligaments were repaired with suture anchors. Fluoroscopy showed that the ankle joint matched well, and no significant signs of secondary dislocation under stress was observed (Fig. 2 a,b). The patient was given plaster external fixation for 6 weeks after surgery, and postoperative analgesia and routine antibiotics were used to prevent infection. Weight-bearing and other vigorous activities were restricted for 10 to 12 weeks after surgery.

**Figure 3:**
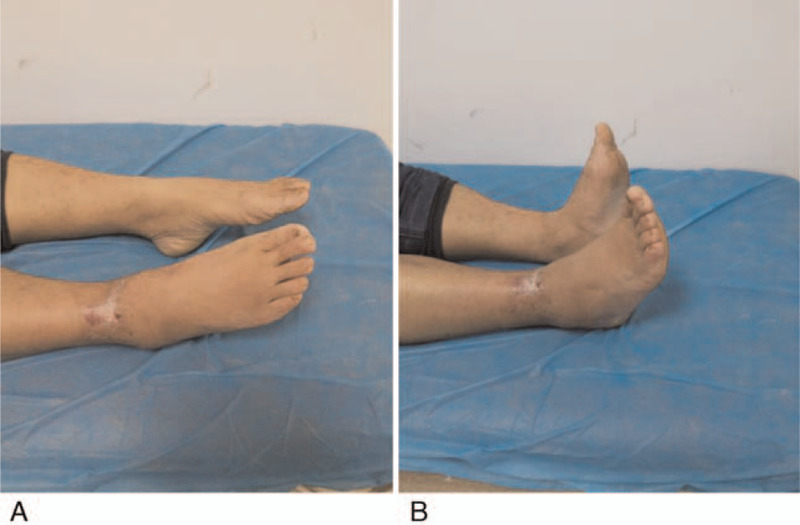
(A, B) Good range of motion of metatarsal flexion and extension.

The open wound healed at 2 weeks after operation, and no complications such as infection and skin necrosis occurred (Fig. 2c). The last follow-up showed a good range of metatarsal flexion and extension of the injured foot, and obvious signs of traumatic arthritis was not observed (Fig. 3). According to Kaikkonen ankle function score, the patient was assessed as 90 points.

## 3 Discussion

Ankle joint is a complex hinge structure in which both bones and ligaments play important and inseparable parts. Complete dislocation of the ankle joint without any fractures is relative rare. The common causes for such injure mainly include dysplasia of medial malleolus, insufficient talus coverage, ligament relaxation, and fibula muscle weakness.^[2]^ Previous studies have reported that the incidence of isolated ankle dislocation approximately is 0.065%, and it is more common in male patients.^[3]^ Actually, sports injuries and traffic accidents are the main causes of such injuries,^[4]^ and forced inversion or eversion caused by high-energy violence is the main injury mechanisms. In the present study, the patient suffered an impact injury, in which his leg was struck anteriorly resulting in the tibia being forcefully pushed posteriorly and then the talus was pushed laterally when the patient twisted his ankle for escape. This mechanism was further confirmed during surgery. Fahey et al^[5]^ has reported 5 common types of ankle dislocation, including anterior dislocation, posterior dislocation, medial dislocation, lateral dislocation and combined dislocation. The patient in our study had a complete lateral dislocation, which is clinically uncommon because the position of lateral malleolus is lower than that of the medial malleolus.

For the treatment of total dislocation of ankle joint, we tried manual reduction firstly but it failed in the early stage. Actually, repeated reduction probably led to more injury in the surrounding soft tissues and increasing pain if the injury mechanism or radiography data has not been obtained. Moreover, if the process of manipulative reduction becomes complicated, the problem of tendon entrapment or dislocation should be considered. Bae et al suggested that the signs of tendon entrapment or dislocation can be diagnosed and excluded by routine CT scan examination, so as to guide the correct steps for treatment.^[6]^ Ballard et al has retrospectively analyzed the radiography data of 398 patients with ankle and hindfoot fractures, and evaluated the incidence of related tendon entrapment and dislocation. In Ballard study, 64 cases (16.1%) with tendon entrapment or dislocation were diagnosed, and the total incidence of tendon entrapment reached up to 8.3%, with tendon dislocation to 8.5%.^[7]^ Therefore, tendon entrapment and dislocation are not uncommon in such complex injuries and should be considered when dealing with ankle dislocation.^[8]^

Since only a few studies have investigated small amounts of patients with conflicting complication rates and no standardized treatment strategy is available, previous studies on the surgical treatment for total dislocation of ankle joint are limited, especially with respect to an issue about whether it is necessary to repair the torn ligament in an early stage.^[9]^ Previous investigators have recommended repair of the lateral ligaments at the time of debridement.^[10,11]^ However, several studies have showed that there was no significant difference in ankle joint function and other related complications between patients with ligaments repaired and those without repair.^[12]^ In the present study, one-stage repair of the torn collateral ligaments were performed and plaster fixation was used for 6 weeks after operation, which increased the probability of ligament healing and maintained the stability of the ankle joint. Weight bearing and functional exercise was carried out gradually, and the final outcome was satisfactory. Nevertheless, we acknowledged that repair of the injured ligaments would increase the operating time and incidence of infection. In addition, just 1 case was involved in our study, hence whether it is necessary for primary repairm of ligaments need more sample size and large randomized controlled trials.

Conclusively, for open total dislocation of ankle joint, urgent reduction of the dislocated ankle and protection of the weak soft tissues are essential. Standardized management such as thorough debridement, wound care and stable temporary immobilization with external fixation can reduce the incidence of related complications.^[13–15]^ However, more clinical researches are needed for such rare trauma to provide more reasonable treatment approach.

## Acknowledgments

The authors would like to thank our department colleagues and the devotion of this patient, and the patient has signed the informed consent form.

## Author contributions

**Conceptualization:** bin wu.

**Data curation:** Hai-bin Wang.

**Formal analysis:** Liang Han, Long-fei Gao.

**Investigation:** Xu Zhang.

**Resources:** Rong-hua Tian.

**Writing – original draft:** Yu-lei Chi, Ying-jie Xu.

**Writing – review & editing:** Xu Gao, Xian-min Bu, bin wu.
